# Supplementary material for: DTI-Voodoo: machine learning over interaction networks and ontology-based background knowledge predicts drug–target interactions
Source: Bioinformatics. 2021 Jul 28;37(24):4835–43. doi: 10.1093/bioinformatics/btab548 (PMC8665763; doi:10.1093/bioinformatics/btab548)
Supplement: btab548_Supplementary_Data [file btab548_supplementary_data.pdf]

DTI-Voodoo: Supplement data

Tilman Hinnerichs and Robert Hoehndorf

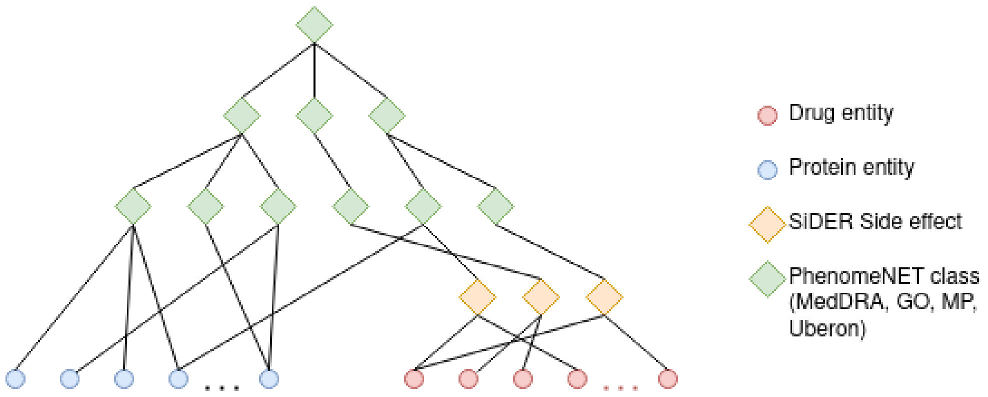

Figure 1: Drugs and proteins with annotations to SiDER and PhenomeNET

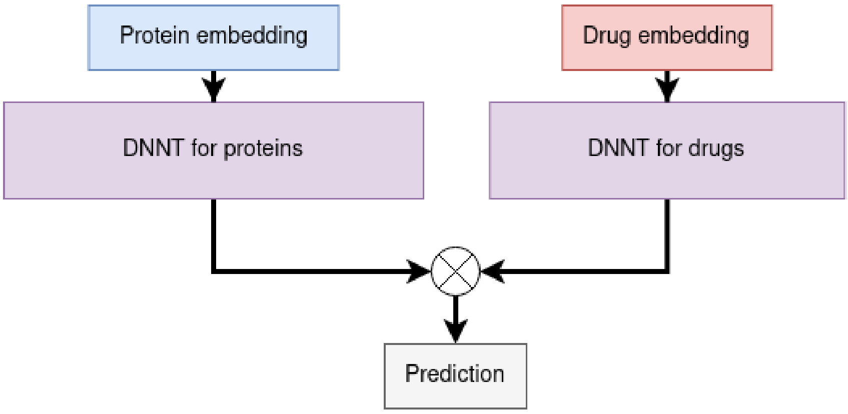

Figure 2: Half-twin network applied to molecular and DL2vec features, utilizing deep learnable feature transformations (LFT). The similarity function $\otimes$ yields the similarity between both transformed embeddings e.g. by computing the cosine similarity.

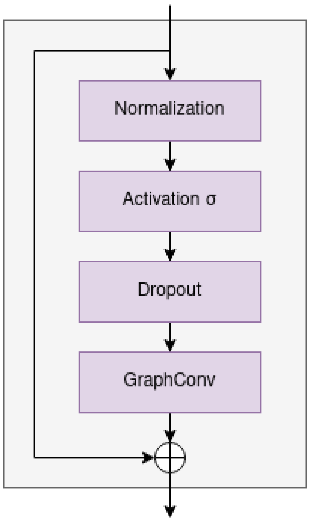

Figure 3: Residual architecture built by *Li et al. (2019, 2020b)* enabling deeper graph convolutional models

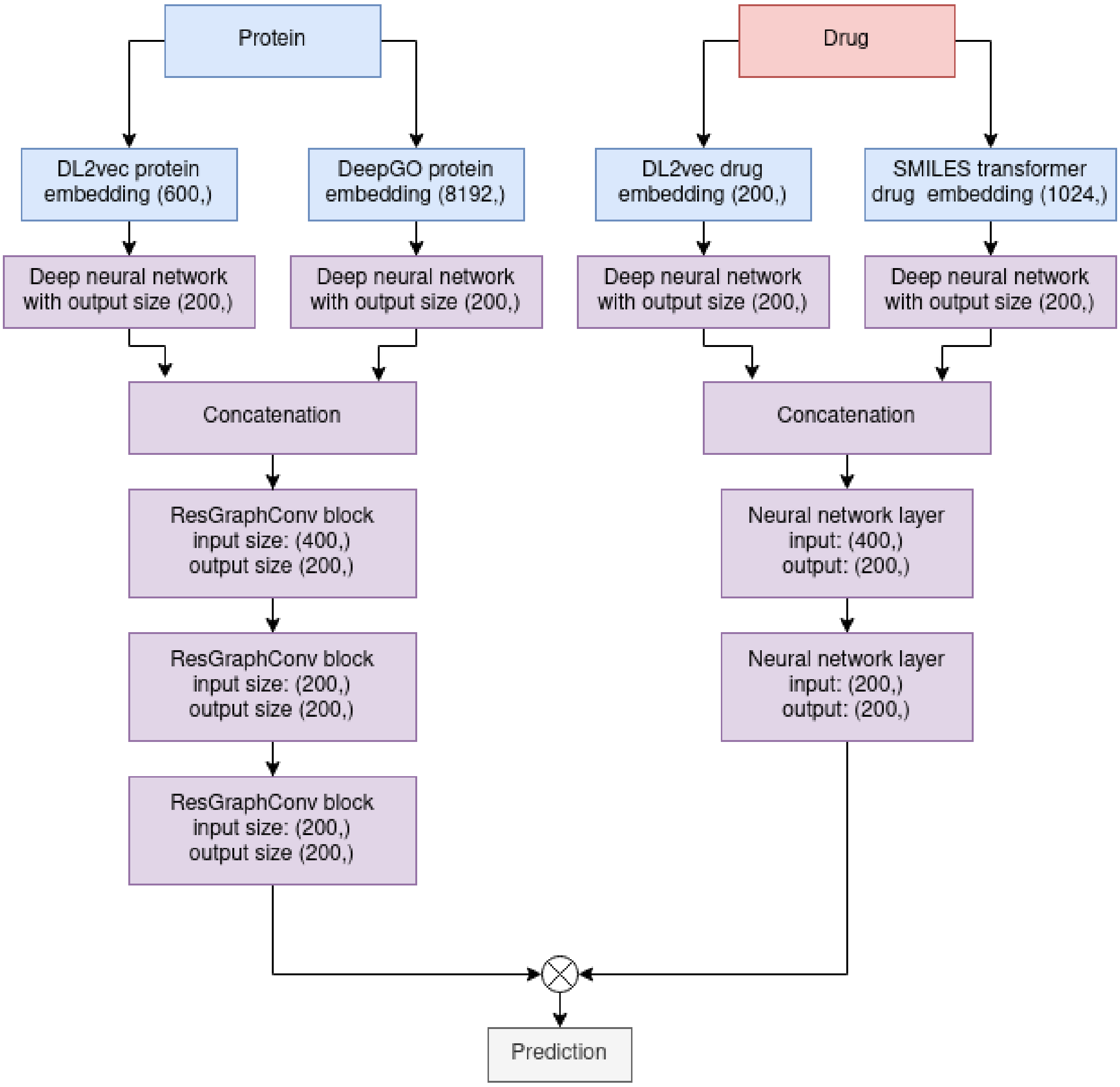

Figure 4: Residual architecture built by *Li et al. (2019, 2020b)* enabling deeper graph convolutional models

	(a) STITCH results					
DTI-Voodoo results	PPI graph					
	without			with		
	Macro AUC	Micro AUC'_p	Micro AUC_p	Macro AUC	Micro AUC'_p	Micro AUC_p
MolPred	0.69	0.66	0.65	0.69	0.68	0.67
OntoPred	0.88	0.89	0.87	0.92	0.94	0.93
DTI-Voodoo	0.89	0.91	0.90	0.93	0.94	0.94

Table 1: Extended results table for MolPred, OntoPred and DTI-Voodoo over STITCH with added $MicroAUC'_p$ (without imputation) for comparison of both metrics.

Approach	Graph convolution method (MacroAUC)					
	without	without/ empty RGC	GCN-Conv	GEN-Conv	RGC + GCN-Conv	RGC + GEN-Conv
MolPred	0.69	0.67	0.67	0.68	0.65	0.69
OntoPred	0.88	0.87	0.88	0.90	0.86	0.92
DTI-Voodoo	0.89	0.89	0.89	0.91	0.86	**0.93**

Table 2: Results for various neural graph convolutional methods enhancing MolPred, OntoPred and DTI-Voodoo over the STITCH dataset. We evaluated all methods over different stacking heights without and with residual graph convolution blocks (RGC, ResGraphConv, see Supplementary figure 3). "Empty RGC" denotes a RGC block with a plain neural layer substituting the GraphConv layer.

	Interaction type			
	Activation		Inhibition	
#Drugs	723		954	
#Proteins	4562		6222	
#Links	14174		16364	
Metric	Macro AUC	Micro AUC_p	Macro AUC	Micro AUC_p
DTI-Voodoo	0.95	0.95	0.93	0.94

Table 3: Extended results table for MolPred, OntoPred and DTI-Voodoo over STITCH with added $MicroAUC'_p$ (without imputation) for comparison of both metrics.

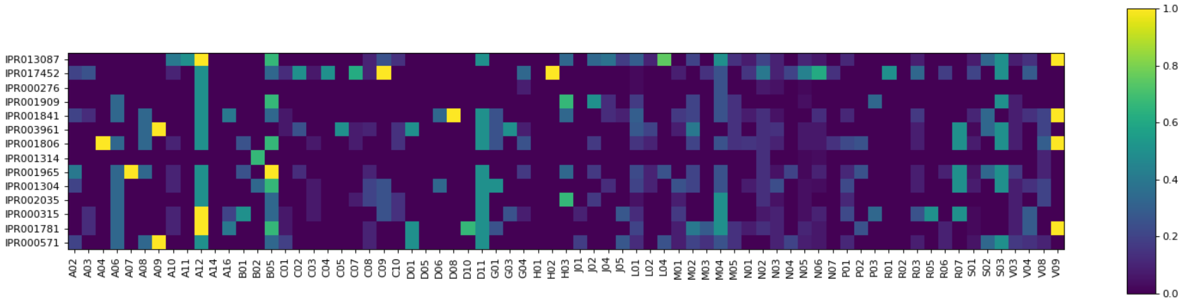

Figure 5: Heatmap showing the results of the ATC drug class and InterPro family prediction analysis. The figure shows the proportion of false negatives compared to the total amount of drugs in the corresponding level 2 ATC class. While few ATC classes show a high share of novel interactors, this is mainly due to the total amount of drugs in the ATC class. For more information note the correspondence with Supplementary Figure 6.

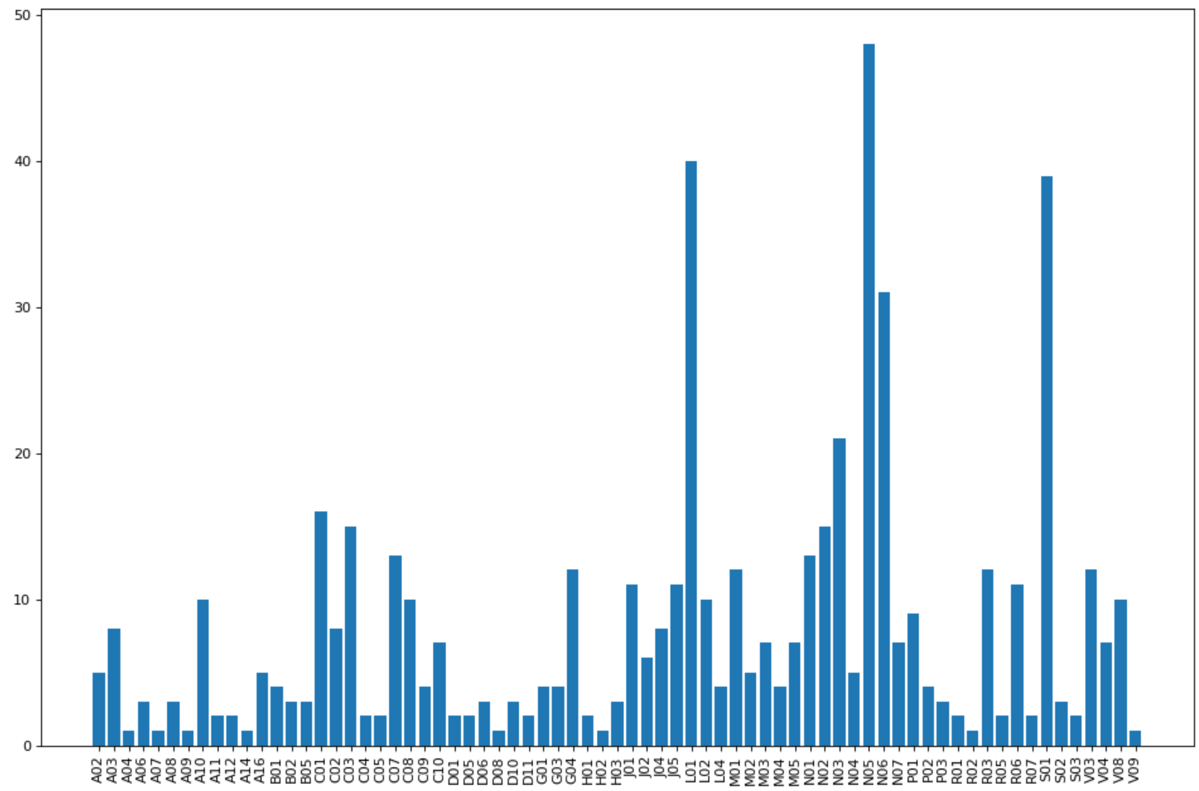

Figure 6: Amount of drugs used in the ATC/InterPro analysis for each level 2 ATC class. Notably, some of the classes have only few or single drug members.
